# Development of Solid Waste-Based Composite Calcium Ferrite Flux and Its Application in Hot Metal Pre-Dephosphorization

**DOI:** 10.3390/ma17050992

**Published:** 2024-02-21

**Authors:** Zheng Zhao, Xiaoming Feng, Yanling Zhang, Yao Zhang, Yaoting Wu

**Affiliations:** 1 State Key Laboratory of Advanced Metallurgy, University of Science and Technology Beijing, Beijing 100083, China; suc_zheng@163.com (Z.Z.);; 2 Jianlong Steel Holdings Co., Ltd., Beijing 100070, China

**Keywords:** compound calcium ferrite flux, melting properties, hot metal pre-treatment, dephosphorization, solid waste disposal

## Abstract

To enhance the slagging efficiency of the lime-based slag system during the pre-treatment stage of hot metal, a composite calcium ferrite flux based on aluminum industry solid waste was developed in this study. The melting characteristics of the flux and its application in the pre-treatment of hot metal were investigated. The results indicated that the main phases of the composite calcium ferrite were CaFe_2_O_4_, Ca_2_Fe_2_O_5_, and Ca_2_(Fe,Al)_2_O_4_. It exhibited high oxidation, high alkalinity, and a low melting point, thereby achieving excellent melting performance. Simulations of various dephosphorization fluxes in the pre-treatment of high-phosphorus hot metal, ordinary hot metal, and kilogram-scale dephosphorization experiment processes were conducted. Under the same experimental conditions, the composite calcium ferrite flux was able to achieve a dephosphorization rate of over 90% and a final phosphorus content of less than 0.02 wt% under high carbon content ([%C] = 3.2 wt%). In the application of hot metal pre-dephosphorization, this flux was able to achieve efficient melting and rapid slagging of lime at a lower temperature, and its slagging time was 50% faster than that of calcium ferrite flux. In addition, this flux enhanced the utilization efficiency of lime during the steelmaking process, effectively prevented the agglomeration of slag, and achieved efficient slag–metal separation. These characteristics were significantly better than the application effect of calcium ferrite flux. This flux has significant implications for the industrial application of deep dephosphorization in the pre-treatment stage of hot metal or the early stage of converter steelmaking.

## 1. Introduction

The pre-dephosphorization of hot metal or efficient dephosphorization in the early stages of converter smelting provided advantages for the production of low-phosphorus steel, while also reducing smelting costs and reducing slag emissions [1,2]. During the early stage of converter smelting, the temperature of the hot metal was low and the activity coefficient of [P] was high, which was thermodynamically very favorable for dephosphorization [3,4]. However, to enhance the efficiency of pre-dephosphorization, it is crucial to create favorable kinetic conditions, namely, the rapid dissolution of lime and the swift formation of a fluid initial slag [5,6]. This is particularly important in cases where the initial [Si] in hot metal is low (in blast furnace ironmaking processes, [Si] is often reduced to below 0.2% or even lower to decrease the fuel ratio) or where there is a high proportion of scrap steel and insufficient heat. The use of effective fluxes that promote lime dissolution becomes crucial. CaF_2_ is an effective flux to promote the dissolution of lime, but due to its potential hazards to the environment and human health, it has been widely banned [7]. Currently, the development of fluoride-free efficient fluxes has received much attention.

Numerous studies have shown that Al_2_O_3_ and Na_2_O significantly promote the dissolution of lime. Diao et al. [8] added small amounts of Na_2_O and Al_2_O_3_ to fluoride-free CaO-FeO-SiO_2_ slag to reduce the melting point and enhance dephosphorization reactions. With only 0.7 to 3.1% w(Na_2_O) in the dephosphorization slag, a dephosphorization rate of 81.4 to 90.7% was achieved. Du et al. [9] found that Na_2_O reduced the activity coefficient of P_2_O_5_ in steelmaking slag and can substitute for CaO in the solid solution to form 2CaO·SiO_2_–Na_2_O·2CaO·P_2_O_5_(C2S-N2C2P), increasing the distribution ratio of P_2_O_5_ in the slag. Our team [10,11] previously proposed using red mud rich in Fe_2_O_3_, Al_2_O_3_, and Na_2_O, generated by the alumina industry, as a steelmaking flux. Semi-industrial trials at the 200 kg scale demonstrated that even at high [%C] contents of 2 to 3 wt%, a dephosphorization rate of over 90% and an endpoint [%P] content of 0.02 wt% were still achieved. However, these substances are not suitable for use in the early stages of converter steelmaking or when the hot metal temperature is low, as their high melting points (>1300 °C) hinder rapid melting under hot metal temperatures. Only after melting and releasing components, such as Fe_2_O_3_, Al_2_O_3_, and Na_2_O, do they effectively promote the dissolution of lime.

Calcium ferrite, an excellent fluoride-free flux, has a low melting point and rapidly forms a highly oxidative and alkaline initial slag [12,13,14]. Sukenaga et al. [15] found that the viscosity of a slag system with 19 wt% CaO and 81 wt% FeO_X_ was 0.065 Pa·S, significantly lower than the 2.4 Pa·S of a CaO-SiO_2_-MgO-Al_2_O_3_ system, suggesting that a more fluid slag system can facilitate phosphorus transfer to the slag. The studies conducted by Jeon et al. [12] and Lee et al. [16] illuminated the synthesis of calcium ferrite melts. Their findings underscored the crucial role of oxygen partial pressure and iron oxide morphology in influencing the formation kinetics of Ca_2_Fe_2_O_5_. Furthermore, they demonstrated that the sintering temperature could be effectively reduced to a range of 1000–1100 °C by incorporating a specific quantity of Al_2_O_3_ into the calcium ferrite, thereby facilitating the formation of Calcium Aluminum Ferrite (CAF). Lee et al. [13] investigated the mechanism and impact of calcium ferrite flux on lime melting, noting that when lime surfaces were coated with iron oxides compared to uncoated samples, more low-melting-point calcium ferrite phases formed, reducing the mechanical properties of lime and making it more easily dissolved in steelmaking slag. Studies by Sato et al. [3] and Mi et al. [17] showed that, due to its low melting point and highly oxidizing capabilities, calcium ferrate was a suitable agent for use in hot metal dephosphorization tests. These tests achieved high dephosphorization rates, nearly 90%, at lower temperatures and in the presence of high levels of [%C]. Wu et al. [1,14] used iron oxide scale and lime-synthesized calcium ferrite in industrial hot metal dephosphorization trials, revealing that calcium ferrite reduced the melting temperature of slag and increased the amount of slag in the double-slag process. The average dephosphorization rate of hot metal with calcium ferrite slag was 88.06%, and the average melting temperature was 1137 °C, superior to the 77.7% dephosphorization rate and 1296 °C melting temperature of fluorspar slag. Moreover, Table 1 summarizes the variety of melts synthesized from solid wastes, along with their respective applications. Generally, these fluxes are classified into non-pre-melting and pre-melting types. The common disadvantage of non-pre-melting fluxes lies in their high melting point, necessitating more heat absorption during the melting process. The disadvantages of pre-melted fluxes include either the high cost of raw materials or the complexity and difficulty of the preparation process. It is anticipated that the use of these fluxes may prove challenging to popularize. Calcium ferrate is considered the best flux to replace CaF_2_ due to its significant advantages in slag forming and hot metal dephosphorization. However, calcium ferrite products are currently rare in the market, mainly due to the high cost of the raw materials (typically fine ore powder and lime) and preparation. Steel producers urgently need to find a flux with similar fluxing effects but at a relatively lower preparation cost.

In this study, red mud, enriched in Fe_2_O_3_/Al_2_O_3_/Na_2_O and produced by alumina companies, was used to develop a flux primarily composed of calcium aluminoferrite. The melting characteristics and deep dephosphorization effectiveness of the new fluxes for the hot metal pre-treatment process were analyzed and compared with conventional lime slagging agents and calcium ferrite fluxes. The aim was to provide significant guidance for the cost-effective industrial application of this novel flux in the pre-treatment of hot metal or the early stages of converter steelmaking.

## 2. Thermodynamic Analysis of Flux Preparation

**Figure 1:**
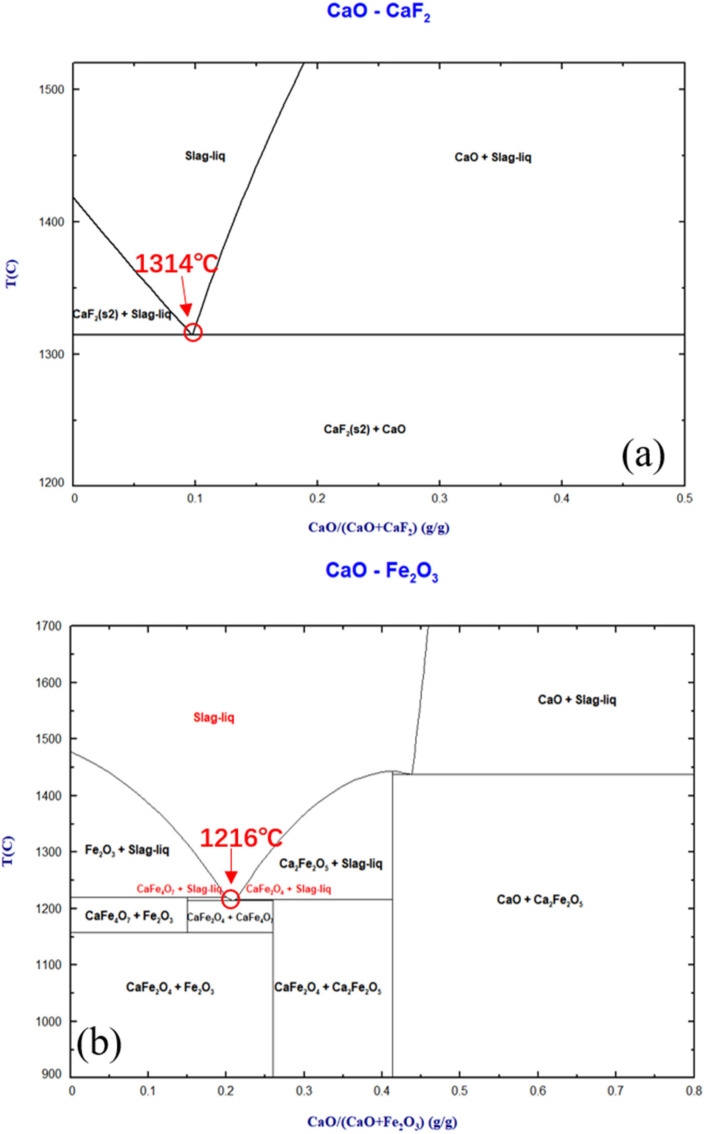

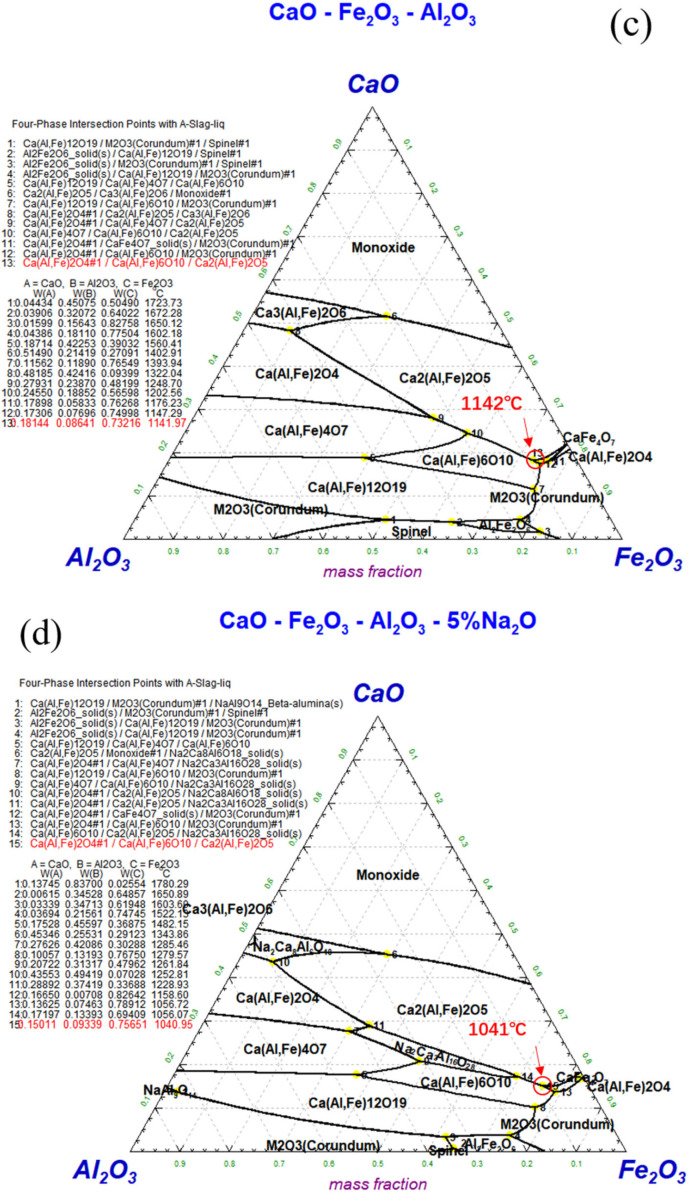
Mineral phase design based on (**a**) CaO-CaF_2_, (**b**) CaO-Fe_2_O_3_, (**c**) CaO-Fe_2_O_3_-Al_2_O_3_, and (**d**) CaO-Fe_2_O_3_-Al_2_O_3_-5%Na_2_O phase diagrams. The yellow circles are added by the software itself and represent intersections between different phases.

The optimal conditions for pre-dephosphorization of hot metal theoretically involve a lower reaction temperature, along with the formation of highly alkaline and strongly oxidizing slag. The development of fluorine-free dephosphorizing fluxes, characterized by a low melting point, high alkalinity, and superior oxidizing capacity, presents an extremely attractive proposition [1,3,5]. Figure 1a–d displays the phase diagrams generated using the thermodynamic software FactSage8.3 for CaO-CaF_2_, CaO-Fe_2_O_3_, CaO-Fe_2_O_3_-Al_2_O_3_, and CaO-Fe_2_O_3_-Al_2_O_3_-5%Na_2_O. CaF_2_ significantly enhances the dissolution of lime primarily because it forms low-melting-point compounds with CaO, with the lowest melting point being 1314 °C [25]. Consequently, the addition of CaF_2_ during the steelmaking process was found to promote the rapid melting of lime. In the CaO-Fe_2_O_3_ phase diagram (Figure 1b), the lowest melting point, at 1216 °C, occurs when CaFe_2_O_4_ forms at a molar ratio of about 1:1 with CaO and Fe_2_O_3_, which is lower than typical hot metal temperatures [12]. Figure 1c shows that adding Al_2_O_3_ to the CaFe_2_O_4_ phase further reduces its melting point to 1142 °C, with a composition of approximately 18%CaO, 9%Al_2_O_3_, and 73%Fe_2_O_3_. Adding Na_2_O to this composition further reduces the melting point to around 1041 °C, as shown in Figure 1d, with the mineral phases being Ca(Al,Fe)_2_O_4_, Ca(Al,Fe)_6_O_10_, Ca_2_(Al,Fe)_2_O_5_, and Slag-liq. Analyzing different systems’ phase diagrams reveals a pattern: the lowest liquid-phase temperature in binary phase diagrams is found in the mineral structures of CaFe_2_O_4_, CaFe_4_O_7_, and Ca_2_Fe_2_O_5_. When new components Al_2_O_3_/Na_2_O are added, isomorphic new solid solutions of calcium ferrite phases such as Ca(Al,Fe)_2_O4, Ca(Al,Fe)_6_O_10_, and Ca_2_(Al,Fe)_2_O_5_ can form, or Na-containing solid solutions and liquid slag phases may develop [9,16,26]. Numerous studies [26] have confirmed that Al^3+^ and Fe^3+^ have similar ionic radii and charge balance, allowing them to occupy the same positions in the lattice without changing the structure. The formation of Na^+^ solid solutions is more related to the substitution of Ca^2+^, as Na^+^ and Ca^2+^ have similar ionic radii, and the substitution does not affect the phase structure [9]. In conclusion, calcium aluminoferrite materials containing Na_2_O have a melting point far below the temperature of hot metal, and as Figure 1c,d shows, these materials contain a considerable amount of Fe_2_O_3_. When added to hot metal along with lime, they can create highly favorable thermodynamic and kinetic conditions for the pre-dephosphorization of hot metal.

## 3. Experimental

### 3.1. Materials

In this study, red mud sourced from an alumina company, which had undergone magnetic separation (Table 2), was used as the raw material. A specific proportion of lime (Table 2) was added and mixed uniformly. After sintering at 1100 °C for 2 h in a muffle furnace, a composite calcium ferrite, referred to as SW-CAF in this paper, was successfully synthesized. Its chemical composition is presented in Table 2. Additionally, following the current standard YBT-4266-2011 for metallurgical CF-65 type physicochemical indicators, laboratory synthesis of calcium ferrite flux (CF-65) was conducted using Fe_2_O_3_ and lime, which were gradually cooled to room temperature after being held at 1250 °C for 2 h, with the composition detailed in Table 1. Both fluxes were prepared under air atmosphere and the heating rate for the ramp-up was set to 8 °C/min. The pig iron used for the dephosphorization experiments was obtained from a specific company, with its composition shown in Table 3. High-phosphorus pig iron was prepared by adding a ferrophosphorus reagent (Table 3) to the base pig iron, as indicated in Table 3. Before the experiments, all materials were dried at 110 °C to a constant weight.

### 3.2. Equipment and Procedures

#### 3.2.1. Melting Temperature Measurement

**Figure 2:**
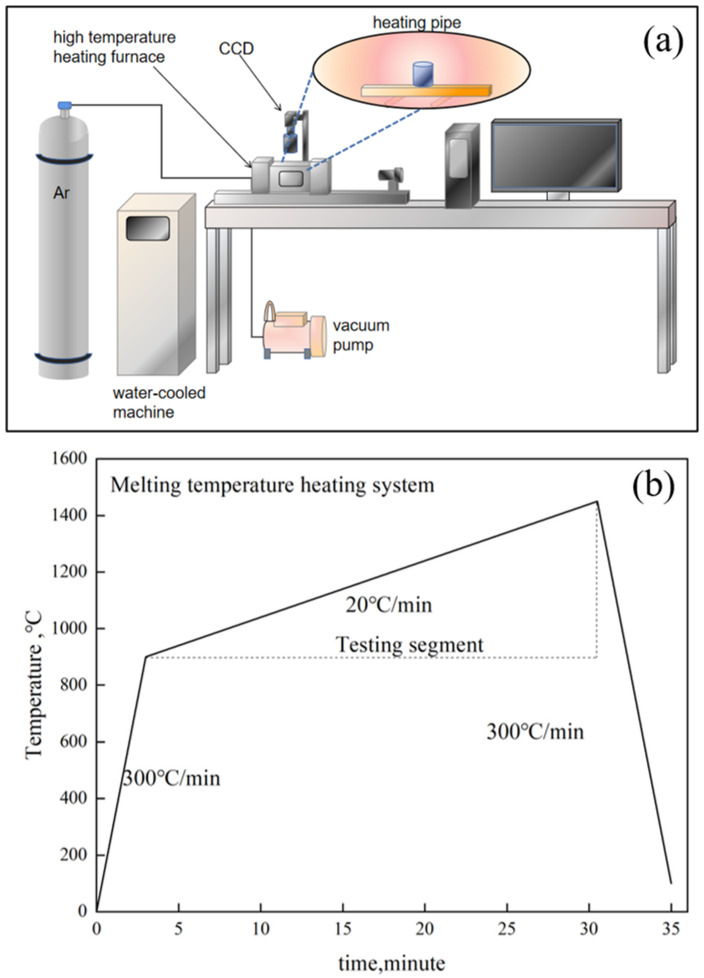

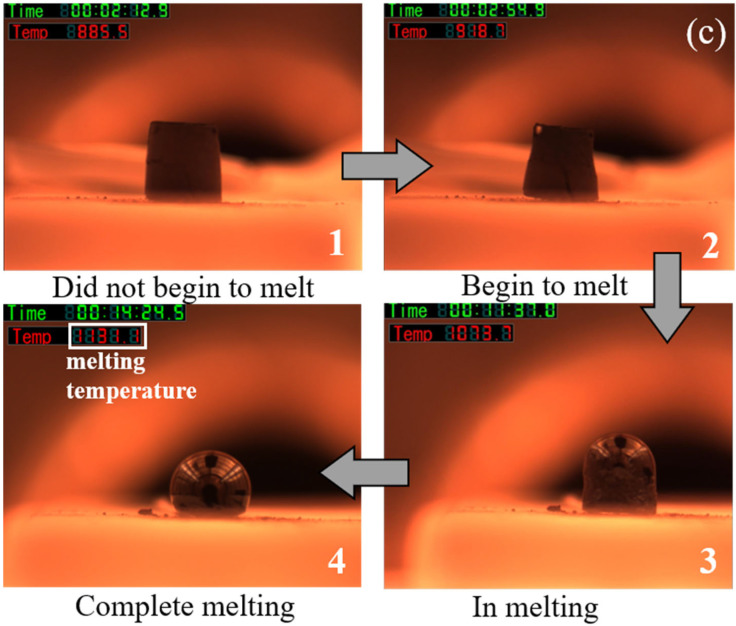
(**a**) The high-temperature reaction in situ observation system, (**b**) the heating regime for melting temperature tests, (**c**) and the testing process of melting temperature. The arrows are guides to the next step and the numbers represent the steps and flow.

The melting points of the synthesized fluxes CF-65 and SW-CAF were tested using a high-temperature reaction in situ observation and online analysis system [27,28], as depicted in Figure 2a–c. This system comprises a three-dimensional super-depth-of-field high-temperature video microscope (CCD), a high-temperature microscope for contact angle measurement, and a high-temperature furnace, equipped with the HiTOS observation and control system for real-time in situ video imaging and data recording. The high-temperature furnace uses a halogen light source for heating, with a maximum temperature rise/fall rate of 1000 °C/min. The procedure was as follows: first, the CF-65 and SW-CAF flux samples were ground to powders of less than 80 μm, then molded into cylindrical shapes of Φ3 mm × H4 mm using a mold, as shown in Figure 2a, and placed on a rectangular MgO piece with dimensions of H1.5 mm. Subsequently, the MgO piece was placed on the furnace chamber rack for heating, as illustrated in Figure 2b. The heating regime during the testing phase controlled the temperature rise/fall rate at 20 °C/min. The melting of the fluxes underwent three stages: overall shrinkage, the beginning of liquid phase formation with solid-liquid coexistence, and complete liquefaction. The melting temperature was determined by the temperature at the initial moment when the height became constant after complete liquefaction, as shown in Figure 2c, corresponding to the fourth step in the material melting process (the temperature is displayed in the upper left corner).

#### 3.2.2. Hot Metal Dephosphorization

(1) Laboratory experiment: The dephosphorization pre-treatment of hot metal was conducted in a MoSi_2_ resistance furnace. Throughout the experiment, argon gas (99.9%) was introduced from the top of the vertical furnace to prevent oxidation of the hot metal and maintain a relatively stable oxygen partial pressure and atmosphere. Initially, the pig iron was heated from room temperature to 300 °C at a rate of 25 °C/min and then to 1410 °C at a rate of 8 °C/min (for the high-phosphorus group, pre-weighed FeP was added to the melt). After maintaining the sample at 1410 °C for 20 min to ensure uniform temperature, a sample weighing 20–25% of the hot metal, approximately 245 g, was added to the surface of the molten metal.
(2) Experiment with a 10 kg induction furnace: To emulate industrial trial conditions, the initial sample addition in the induction furnace was determined to cover the surface of the molten steel, approximately 13% of the hot metal weight. During the experiment, 2.3 kg of pig iron was heated in a magnesia crucible from room temperature to 1410 °C. Once the hot metal was completely molten, the temperature was held constant for 5 min to collect initial samples. Under three experimental conditions, mixtures of (CaO+Fe_2_O_3_), (CaO+Fe_2_O_3_+CF), and (CaO+Fe_2_O_3_+CAF) were added to the surface of the molten metal and reacted for 5 min before collecting steel and slag samples. The reaction continued for additional periods of 10, 15, and 20 min, after which steel and slag samples were collected, and the steel was cast at high temperatures.

Post-experiment, the crucibles containing the quenched samples were placed in an oven to completely dry. The slag and pig iron were physically separated for subsequent sample analysis.

#### 3.2.3. Experimental Scheme of Pre-Dephosphorization

The experimental scheme is presented in Table 4. It includes trials H1 to H3 and L1 to L3, which utilize high-phosphorus iron with an initial phosphorus content (P0) of approximately 0.25 wt% and regular iron with a P0 of about 0.15 wt%, respectively, for preliminary dephosphorization laboratory tests. Experiment G3, using standard pig iron and conducted in a 10 kg induction furnace, follows the same protocol as H3 and L3. Its purpose is to further assess the efficacy of SW-CAF in the preliminary dephosphorization of hot metal. In experiments H1 and L1, no flux is added; lime and Fe_2_O_3_ are the sole dephosphorization agents. Other trial groups are formulated by adding various types of flux to the CaO and Fe_2_O_3_ base. It is important to note that in all experiments, the composition of the dephosphorization agent remains constant, with the total mass ratios of CaO to Fe_2_O_3_ being identical.

### 3.3. Analysis

The structures, the prepared materials, and the slag samples were characterized by using an X-ray diffract meter (XRD, Rigaku, SmartLab, Tokyo, Japan). The composition of the samples was analyzed using X-ray fluorescence spectroscopy (XRF, PANalytical, AXIOSmAX, Almelo, Netherlands). A 1 g metal sample was used to determine the [C] content with a carbon/sulfur analyzer (CS, Eltra, CS-800, Haan, Germany). A 0.1 g sample was completely dissolved in a solution of 3 mol/L HCl and 1 mol/L HNO_3_, and the [P] content was then determined using an Inductively Coupled Plasma Mass Spectrometer (ICP-MS, Thermo Scientific, ICAP RQ, Waltham, MA, USA). The microstructure and properties of the dephosphorized slag were determined by scanning electron microscope energy dispersive spectrometry (SEM-EDS, FEI, MLA250, Hillsboro, OR, USA).

## 4. Results

### 4.1. Phase and Melting Properties of CF and SW-CAF

**Figure 3:**
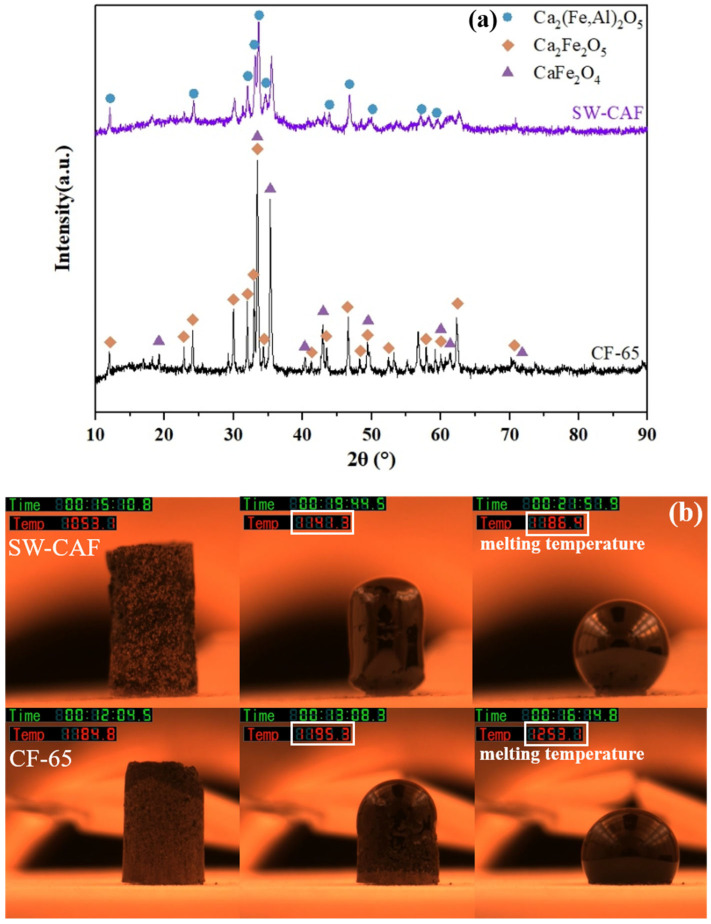
(**a**) XRD patterns and (**b**) melting characteristics of different fluxes.

Figure 3a,b, respectively, display the XRD phases and melting characteristics of two types of fluxes. Figure 3a revealed that the synthetic calcium ferrite flux CF-65 was primarily composed of CaFe_2_O_4_ and Ca_2_Fe_2_O_4_. These mineral phases had low melting points and were characterized by high oxidizing properties and high alkalinity. The predominant phases in SW-CAF were CaFe_2_O_4_, Ca_2_Fe_2_O_4_, and Ca_2_(Fe,Al)_2_O_4_. In addition to the calcium ferrite mineral phases, this flux also contained calcium iron aluminate phases, collectively referred to as composite calcium ferrite phases.

As shown in Figure 3b, the melting temperatures of both fluxes were below 1300 °C, which was sufficient to achieve a complete liquid phase under the temperature requirements for desiliconization and dephosphorization of hot metal (1250~1450 °C). The melting temperature of flux CF-65 stood at 1253 °C, while that of SW-CAF was 1186 °C. During the high-temperature melting process, CF-65 started deforming at 1184 °C, transitioned from a solid to a flowing liquid phase at 1195 °C, and took 186 s to fully liquefy. In contrast, SW-CAF began deforming at 1053 °C, initiated the transition at 1141 °C, and required 147 s to fully liquify. In summary, the melting temperature of the composite calcium ferrite was significantly lower than that of the synthetic calcium ferrite, melting more rapidly and showing superior fluidity. This rendered it suitable as a flux in the pre-treatment stage of hot metal.

### 4.2. Dephosphorization Test Results

**Figure 4:**
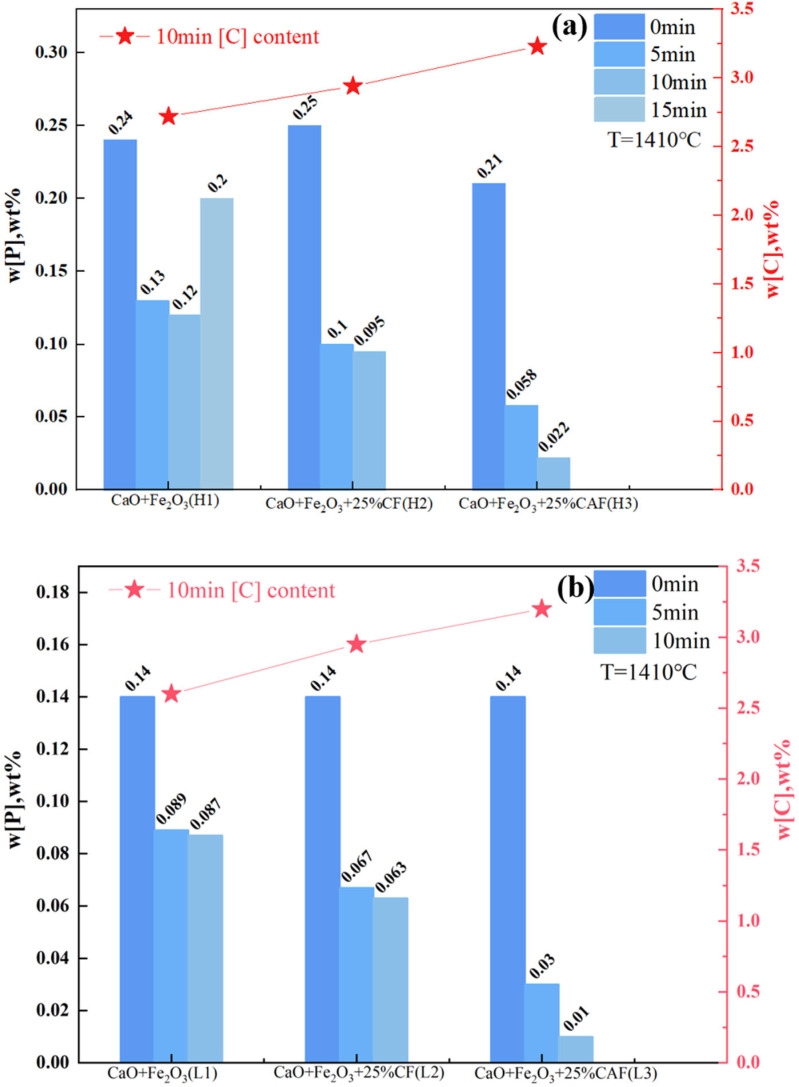
Dephosphorization effects of different flux types under (**a**) high-phosphorus and (**b**) regular iron conditions.

Under high-phosphorus and regular iron conditions, three types of slag materials were used for the pre-dephosphorization of hot metal: CaO+Fe_2_O_3_, CaO+Fe_2_O_3_+25%CF, and CaO+Fe_2_O_3_+25%CAF. The variations in the [P] and [C] contents in the metal over time are shown in Figure 4a,b. It was evident that, under similar temperatures and slag-making regimes, slags containing composite calcium ferrite demonstrated significant dephosphorization effectiveness, followed by those containing calcium ferrite, both surpassing the conventional CaO+Fe_2_O_3_ slag system. This trend was consistent across different initial [%P] contents in the hot metal. Specifically, with high initial phosphorus, the [%P] content in the CaO+Fe_2_O_3_ slag only reduced from 0.25 wt% to 0.12 wt% within 10 min, while the final [%P] in the CaO+Fe_2_O_3_+25%CF slag lowered to 0.095 wt% from 0.25 wt%. The composite calcium ferrite slag reduced [%P] from 0.21 wt% to 0.022 wt% within a short 10 min span, significantly lower than the first two. When the initial [%P] was around 0.14 wt%, after 10 min of dephosphorization, the final phosphorus contents in the three slag systems were 0.087 wt%, 0.063 wt%, and 0.01 wt%, respectively, with the composite calcium ferrite slag achieving the lowest final phosphorus content.

Notably, when the phosphorus content in the hot metal was high, the CaO+Fe_2_O_3_ slag experienced severe rephosphorization at 15 min, attributed to a rapid depletion of FetO, leading to a rise in the slag melting point and reduced fluidity. The CF-containing slag system did not exhibit rephosphorization between 10 and 15 min but lost its dephosphorization capacity, achieving a maximum dephosphorization rate of only 62%. The flux in this study maintained a strong dephosphorization ability even after 10 min, reaching a dephosphorization rate of 90%. This was attributed to components such as Al_2_O_3_/Na_2_O in the slag, which maintained good fluidity even with reduced FetO concentration.

The [C] content during the iron dephosphorization stage was crucial. The CaO+Fe_2_O_3_ slag system exhibited the highest [C] consumption, while the CAF-containing slag had the lowest, with a final [C] content of 3.2 wt%. This study effectively achieved “dephosphorization while preserving carbon,” attributed to the slag system’s superior dephosphorization kinetics under low-temperature conditions, thus enhancing thermodynamic dephosphorization. In the CaO+Fe_2_O_3_ slag system, when dephosphorization kinetics were poor, [P] accumulated at the slag–metal interface and could not diffuse rapidly, creating a local equilibrium. Under these conditions, (FeO) in the slag tended to react with [C], consuming the [C] in the hot metal. The flux in this study successfully achieved deep dephosphorization under high [C] conditions, which was significant for the pre-treatment stage of hot metal or early in converter steelmaking.

**Figure 5:**
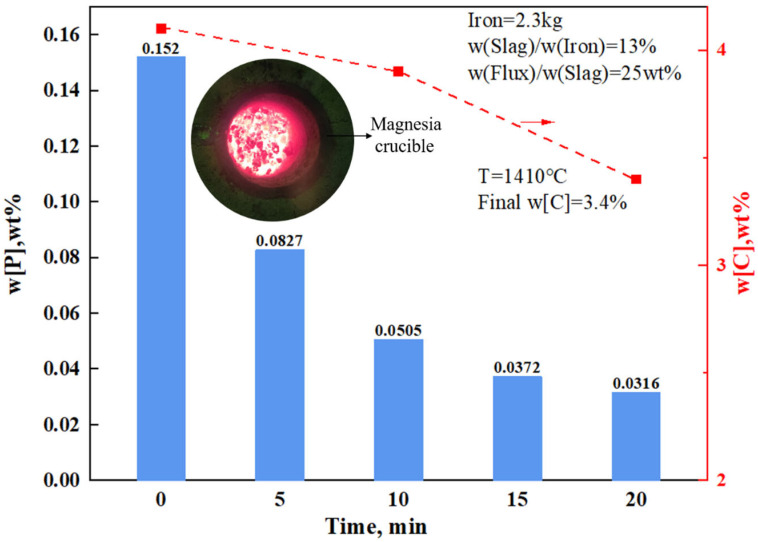
A kilogram-scale experiment.

Figure 5 shows the pre-dephosphorization experiment (G3) in a 10 kg induction furnace. The hot metal temperature was 1410 °C, and under conditions without stirring or oxygen blowing, the initial [%P] was 0.152 wt%. After 20 min, the [%P] in the metal was 0.0316 wt%, achieving a dephosphorization rate of 79.2%, without any rephosphorization. Compared to the initial [%P] of 0.14 wt% in the laboratory experiments, the difference in dephosphorization after 10 min, resulting in the [%P] of 0.01 wt%, was primarily due to varying slag amounts and significant differences in slag surface temperatures. After adding the slag, it took approximately 3 min to form, and the slag still exhibited some fluidity after 20 min of the dephosphorization reaction. The final slag contained 4.7 wt% P_2_O_5_ and had a binary basicity of about 3.2 (Table 5). Despite the deeper melt pool, lower surface temperatures, and reduced slag amount—factors generally unfavorable for dephosphorization—the process still achieved a final phosphorus content below 0.04 wt% within 15 min, meeting the requirements for most plain carbon steel productions. Thus, the experiment overall replicated the results of the low melting temperature and high dephosphorization rate characteristic of composite calcium ferrite flux.

## 5. Discussion

### 5.1. The Slag Formation Process of Different Fluxes

**Figure 6:**
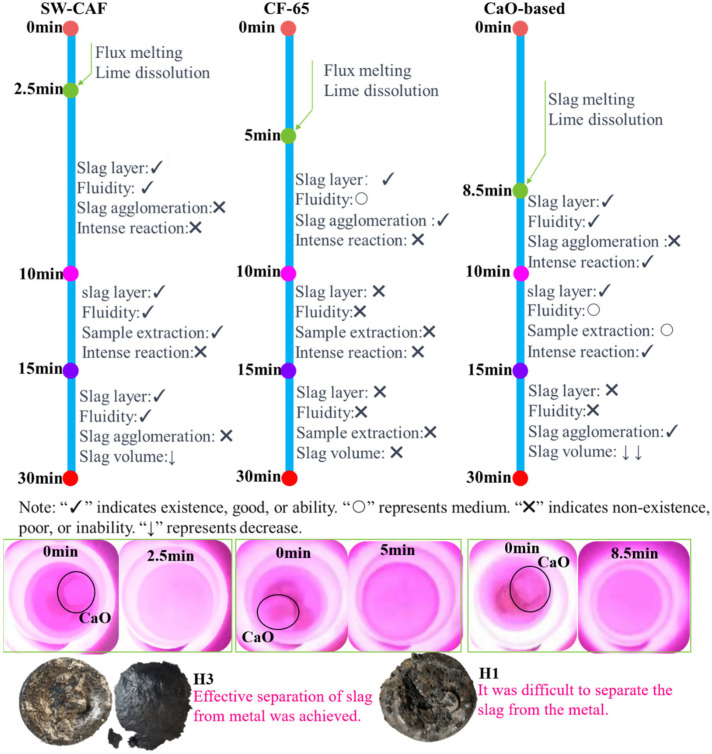
The melting phenomena of different slagging materials.

Figure 6 depicts the slag formation within 30 min during the experiment for three different slag systems under the same hot metal conditions. All lime briquettes, Fe_2_O_3_ briquettes, and flux particles maintained the same size at the beginning and were simultaneously added to the surface of the hot metal. Regarding lime dissolution time, in the slag with added composite calcium ferrite flux, the lime dissolved completely in the shortest time, taking only 2.5 min. In contrast, in the CaO+Fe_2_O_3_ slag system without any added flux, the lime took 8.5 min to fully disappear. Compared to the conventional slag system, the slag formation time with the addition of the flux in this study was over 70% faster.

Regarding the relationship between slag formation and dephosphorization efficiency, during the dephosphorization process in the CaO+Fe_2_O_3_ slag system, the slag became completely liquid 8.5 min after adding the slagging agent. At 10 min into the reaction, slag–metal reaction behavior manifested as intense splashing. As depicted in Figure 4a, for the H1 group at 10 min, the phosphorus content in the metal ([%P] = 0.12 wt%) was relatively high, indicating poor dephosphorization due to the short time that lime participated in the reaction after dissolving, and rephosphorization occurred in the latter 15 min. For the slagging agent containing CF flux, the calcium ferrite flux melted within 5 min and quickly completed melting, after which CaO was immediately dissolved in the liquid slag. At 10 min into the reaction, a small amount of precipitate was observed in the slag, and fluidity began to decline. As shown in Figure 4a, for the H2 group at 10 min, the phosphorus content ([%P] = 0.095 wt%) was slightly lower than the H1 group, attributed to increased slag reaction time after slag formation. However, after 15 min, the slag layer had disappeared, and agglomeration and clumping occurred, a phenomenon referred to as “re-drying”. For the slagging agent containing CAF flux, the flux melted into liquid slag within 2.5 min, followed by rapid dissolution of CaO in the liquid slag. At 10 min into the reaction, the slag–metal reaction continued, with a visible flowing slag layer. As shown in Figure 4a, for the H3 group at 10 min, [%P] had already reduced to 0.022 wt%. The advantage of this flux was in prolonging the re-drying period, with almost no slag-state re-drying, thereby enhancing dephosphorization efficiency.

It was noteworthy that the early dephosphorization effects within 5 min for groups H1 to H3, as shown in Figure 4a, where the [%P] for group H3 (using composite calcium ferrite flux) was significantly lower at 0.058 wt% compared to the H1 and H2 groups. This demonstrated the advantage of the flux’s pre-melting and early slag formation, indicating that a lower melting point leads to faster early slag formation, thus advancing the dephosphorization phase. The dephosphorization pattern was consistent with that of high-phosphorus hot metal.

In discussing the relationship between slag formation, the slag–metal separation interface, and the carbon content, when the dephosphorization process continued for 30 min, the slag with CAF flux, despite the reduced volume, maintained good fluidity. The fluidity of the slag system containing CF flux significantly decreased, introducing complexities to the sampling task. For the CaO+Fe_2_O_3_ slag system, the slag was re-dried, reducing the fluidity of the steel. As depicted in Figure 4a and Figure 6, the steel block from group H3, subsequent to water quenching, exhibited a smooth surface, suggestive of a more comprehensive slag–metal separation. In contrast, the steel block from group H1 presented an uneven surface, which hindered effective slag–metal separation, akin to the observations in the H2 group. Considering the coverage of the slag layer over the steel during the reaction, the final [C] content for the H3 group was 3.23 wt%, while it was 2.72 wt% and 2.94 wt% for the H2 and H1 groups, respectively. In conclusion, the slag with added composite calcium ferrite flux had rapid slag formation, efficient lime dissolution, and good slag–metal separation, all of which played a crucial role in enhancing deep dephosphorization capability. Moreover, the presence of multiple components such as Al_2_O_3_/Na_2_O/TiO_2_ in the flux maintained good fluidity even with substantial consumption of FeO, proving its efficacy as an excellent dephosphorization flux [8,11].

### 5.2. Mineral Phases of Composite Calcium Ferrite Slag

**Figure 7:**
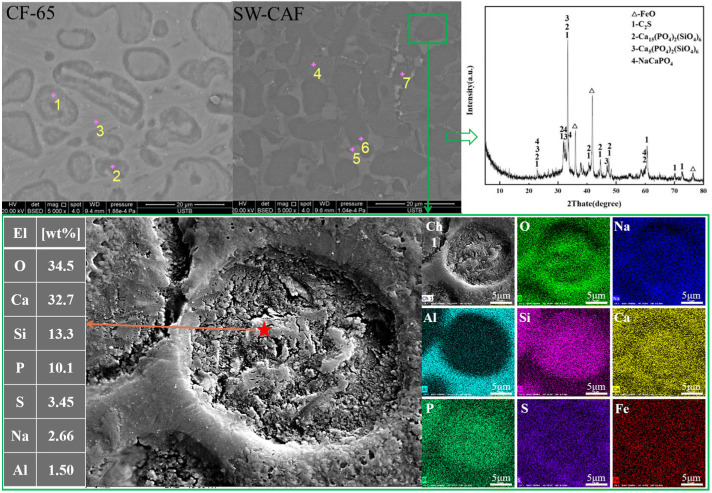
The SEM patterns of CF-65 and SW-CAF final slag, and the XRD pattern of SW-CAF. 1–7 refers to the EDS component information at the corresponding position in the figure. As shown in Table 6.

The micro-morphology and chemical composition of the dephosphorization final slag in the CF and CAF flux-containing slag systems are displayed in Figure 7 and Table 6. SEM-EDS comparative analysis of the final slag phases showed that the CF slag system primarily consisted of unreacted lime particles, dicalcium silicate (C2S), minor calcium ferrite (CF), and solid solution 2CaO·SiO_2_–3CaO·P_2_O_5_(C2S-C3P). The CAF slag system distinctly featured an amorphous matrix phase (gray), iron-bearing matrix phase (white), and oval-shaped phosphorus-enriched phase (dark gray). Combined with XRD phase analysis from Figure 7 for the CAF slag system, the amorphous matrix phase was mainly a low-melting liquid slag phase, the iron-bearing matrix phase was primarily iron aluminate spinel, and the phosphorus-enriched phase existed in the forms of C2S-C3P or nC2S-C3P and Na-rich phosphorus phase N2C2P. Further high-resolution SEM analysis of local slag phases revealed that the phosphorus-enriched phase was concentrated inside the oval shapes, coinciding with the enrichment of elements like Ca, Si, and Na. Particularly, the Na element was enriched around the P element, consistent with the Ca distribution but differing from Si, also proving the formation of the Na-rich phosphorus phase. Compared to the phosphorus-enriched phase area ratio in both slag systems, the proportion in the CAF slag system was higher than in the CF system, with the highest phosphorus content in the solid solution reaching 17.71 wt% (Table 6).

The addition of CAF flux containing Al_2_O_3_/TiO_2_/Na_2_O also impacted phosphorus enrichment. Studies indicated [9] that Al_2_O_3_ could increase the phosphorus content in the C2S-C3P solid solution but also increase the liquid slag phase. When Na_2_O replaced CaO, it could transform from C2S-C3P to C2S-N2C2P, releasing CaO, which then combined to form new C2S, thereby increasing the overall solid solution content in the slag. Hence, under the combined interactions, a phosphorus-enriched phase with a high phosphorus concentration was obtained [8,11].

### 5.3. Utilization Rate of Lime

The utilization rate of lime reflects the performance of the flux. Research by Shiro [5] showed that when [Si] and [Mn] in hot metal are in trace amounts, a dephosphorization agent with over 50% iron oxide content has sufficient oxidizing capability. Therefore, the dephosphorization ability depends on the supply of CaO. In this experiment, the theoretical impact of [Si] and [Mn] in hot metal on the demand for oxidizers was considered. With an iron oxide content above 60% being sufficient, it is still possible to compare the dephosphorization abilities of fluxes using Equation (1).

Δn_P_/nCaO = (n_P0_ − n_Pt_)/nCaO (1)

n_P0_: the initial molar number of [P] in hot metal (mol); n_Pt_: the molar number of [P] in hot metal at *t* minutes (mol); nCaO: the number of moles of CaO added at *t* minutes (mol). Δn_P_/nCaO is defined as the CaO utilization efficiency during hot metal treatment.

**Figure 8:**
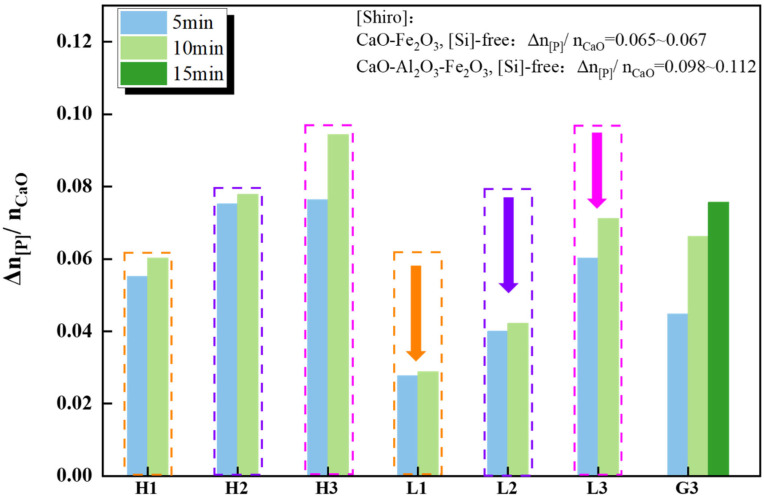
Lime utilization efficiency. Dashed lines of the same color represent comparisons of values using the same melt group. The arrows point to a decreasing trend in values between the same groups.

Figure 8 shows the CaO utilization efficiency in high-phosphorus iron dephosphorization experiments H1 to H3, regular iron dephosphorization experiments L1 to L3, and induction furnace experiment G3. It was observed that under the same [%P] conditions in hot metal, the lime utilization efficiency was highest in the slag system containing CAF flux and lowest in the CaO+Fe_2_O_3_ slag system. Compared to high-phosphorus iron, regular iron generally had a lower lime utilization rate during dephosphorization, even lower than the CaO+Fe_2_O_3_ slag system in high-phosphorus iron. The lime utilization efficiency in the group containing CAF flux (including kilogram-scale expanded experiments) increased over time, while it remained relatively unchanged over time in the group containing CF flux. It was foreseeable that with the increase in reaction time, the advantages of CAF would become more apparent. Comparing the results of this study with Shiro’s research [5], the CaO-Fe_2_O_3_ slag system had similar lime utilization efficiency to CF in this study, while the CaO-Fe_2_O_3_-Al_2_O_3_ slag system’s efficiency was closer to this study’s Al_2_O_3_/Na_2_O/TiO_2_ composite calcium ferrite flux. Since there was no silicon in the hot metal, and no SiO_2_ in the slag, the dissolved lime was primarily used for dephosphorization. In this study, lime was used not only for dephosphorization but also in the formation of calcium silicate, explaining its relatively lower utilization rate. In summary, CAF prepared from solid waste exhibited high efficiency in the pre-dephosphorization of hot metal. This flux not only significantly reduced raw material costs but also enhanced the utilization efficiency of lime, potentially bringing transformative breakthroughs in the pre-treatment phase of hot metal or early converter steelmaking.

## 6. Conclusions

This study, utilizing industrial solid waste red mud, successfully developed a novel low-melting-point flux SW-CAF. The melting characteristics of SW-CAF were explored, and high-temperature dephosphorization experiments were conducted. By comparing the dephosphorization effects of different fluxes, SW-CAF demonstrated excellent lime dissolution and good fluidity, offering significant advantages in the pre-dephosphorization process of hot metal. The specific results are as follows:

(1) The primary phases of the low-melting-point dephosphorization flux were CaFe_2_O_4_, Ca_2_Fe_2_O_4_, and Ca_2_(Fe,Al)_2_O_4_, collectively termed composite calcium ferrite. The complete melting temperature of this flux was about 1186 °C, with the beneficial dephosphorization components (Fe_2_O_3_+CaO) exceeding 80%, offering significant advantages such as a high iron oxide content, high alkalinity, a low melting point, and good melting fluidity.
(2) Simulating the dephosphorization process of hot metal using high-phosphorus ore ([%P] = 0.25 wt%) and regular iron ([%P] = 0.14 wt%), composite calcium ferrite exhibited superior dephosphorization effectiveness compared to synthetic calcium ferrite. With a high carbon content ([%C] = 3.2 wt%), a dephosphorization rate above 90% and a final [%P] content below 0.02 wt% were achieved. Regardless of the initial phosphorus content and the quantity of hot metal (200 g and kilogram-scale), excellent dephosphorization results were replicated using composite calcium ferrite.
(3) In the dephosphorization slag with added composite calcium ferrite flux, the phosphorus-enriched phase was primarily nC2S-C3P solid solution, with a minor presence of Na_2_O-containing N2C2P solid solution. The highest phosphorus content in the solid solution reached 17.71 wt%, significantly exceeding the 3.92 wt% in CF slag.
(4) In the pre-dephosphorization of hot metal, using composite calcium ferrite flux enabled efficient lime melting and rapid slag formation even at lower temperatures. Compared to CF flux, the slag melting time increased by 50% and by 70% compared to the conventional CaO+Fe_2_O_3_ slag system. During dephosphorization, composite calcium ferrite enhanced lime utilization efficiency, extended the re-drying period, and achieved a high dephosphorization rate with lower carbon loss, significantly surpassing CF flux.
(5) At the conclusion of the steelmaking process, the application of composite calcium ferrite flux was observed to facilitate effective slag–metal separation. This is critically significant for the removal of high-phosphorus slag and ensuring efficient slag–steel separation during the final phases of steelmaking.

Consequently, the composite calcium ferrate derived from solid waste is expected to be a superior fluorine-free flux for steelmaking. This innovative material is positioned to play a crucial role in the manufacturing of high-end steel grades with low or ultra-low phosphorus content.

## Figures and Tables

**Table 1:** Fluxes prepared from solid wastes and their applications.

Solid Waste Material	Composites	Preparation Methods	Properties	Refs.
Red Mud	Flux	Room temperature, red mud as an additive	Dephosphorization, desulfurization	[10,11,18]
Red Mud	Flux	Room temperature, briquette of red mud mixed with lime powder	Dephosphorization and desiliconization	[5,19]
Red Mud	Flux	High-temperature pre-reduction, red mud mixed with graphite powder	Desulfurization	[20,21]
Red Mud	Flux	High-temperature roasting, red mud mixed with iron ore powder	Metallurgical properties (reducibility, strength, etc.)	[22,23]
Mill scales	Calcium ferrate flux	High-temperature roasting, mill scales mixed with lime	Dephosphorization	[1,14]
Desulfurization gypsum	Calcium ferrate	High-temperature roasting, desulfurization gypsum, graphite powder mixed with iron ore powder	Desulfurization, sintering	[24]

**Table 2:** Experimental raw material composition (wt%).

Material	Fe_2_O_3_	Al_2_O_3_	CaO	SiO_2_	TiO_2_	Na_2_O	K_2_O	LOI
Red mud	75.5	13.6	0.49	2.49	4.64	2.06	0.02	15.35
SW-CAF	50.74	10.29	30.46	1.76	3.46	1.38		
CF-65	68	1.3	23	2.5				
Lime(AR)			99.8					
Fe_2_O_3_(AR)	99.8							

**Table 3:** Iron initial composition (wt%).

Material	C	Si	Mn	P	S
Pig iron	4.10	0.40	0.32	0.14	0.04
FeP(AR)	0.13	0.38	1.07	21.49	0.06
High phosphorus pig iron	4.10	0.40	0.32	0.25	0.04

**Table 4:** The experimental scheme of the hot metal pre-dephosphorization process (wt%).

No.	Slag Material				[P]_0_ in Hot Metal	Experimental Equipment
	CaO	Fe_2_O_3_	CF-65	SW-CAF		
H1	15.1%	84.9%			0.21~0.25	MoSi_2_ furnace
H2	13.7%	61.3%	25%			
H3	13.7%	61.3%		25%		
L1	15.1%	84.9%			0.14	
L2	13.7%	61.3%	25%			
L3	13.7%	61.3%		25%		
G3	13.7%	61.3%		25%	0.15	Induction furnace

**Table 5:** The final slag composition (wt%).

Slag	CaO	SiO_2_	Fe_2_O_3_	Al_2_O_3_	TiO_2_	Na_2_O	P_2_O_5_
G3	32.9	10.1	34.5	6.11	2.24	0.741	4.70

**Table 6:** The EDS energy spectrum analysis of each point (wt%).

Point	Ca	Si	P	S	Na	Fe	Al	Ti	Mn
1	34.62								
2	40.1	12.23				6.02			
3	64.22	22.8	3.92	1.5					4.57
4	28.94	5.36	2.73	1.85	0.27	5.74	13.85	0.25	5.24
5	28.78	3.48	3.79	1.66	1.06		3.77	12.97	
6	57.9	13.47	17.71	4.19	1.72			6.74	
7	27.72	11.43	9.12	1.87		2.8	7.06		3.51

## Data Availability

Data are contained within the article.
